# A new scoring system facilitating diagnosis of oral squamous malignancy on biopsy specimens

**DOI:** 10.1186/s12903-022-02188-0

**Published:** 2022-05-06

**Authors:** Cheng-Lin Wu, Cheng-Chih Huang, Shang-Yin Wu, Shih-Sheng Jiang, Fang-Yu Tsai, Jenn-Ren Hsiao

**Affiliations:** 1 Department of Pathology, National Cheng Kung University Hospital, College of Medicine, National Cheng Kung University, Tainan, Taiwan; 2 Institute of Clinical Medicine, College of Medicine, National Cheng Kung University, Tainan, Taiwan; 3 Department of Otolaryngology, National Cheng Kung University Hospital, College of Medicine, National Cheng Kung University, 138 Sheng Li Road, Tainan, 70456 Taiwan; 4 Department of Oncology, National Cheng Kung University Hospital, College of Medicine, National Cheng Kung University, Tainan, Taiwan; 5 National Institute of Cancer Research, National Health Research Institutes, Zhunan Town, Miaoli County Taiwan

**Keywords:** Oral squamous malignancy, Oral squamous cell carcinoma (OSCC), Oral verrucous carcinoma (OVC), Immunohistochemistry (IHC), Biomarker

## Abstract

**Background:**

Morphological evaluation of oral mucosal biopsy is sometimes inconclusive, which may delay the diagnosis and treatment of oral squamous malignancy. Immunohistochemical biomarkers denoting oral squamous malignancy would be clinically helpful in such scenario.

**Methods:**

We first studied the expression patterns of four potential biomarkers (cytokeratin 13, cytokeratin 17, Ki-67 and laminin 5 gamma 2 chain) in an exploratory cohort containing 54 surgical specimens from confirmed oral squamous malignancies. A pattern score was assigned to each specific expression pattern of these four biomarkers. A total score from each specimen was then calculated by summing up the four pattern scores. A cut-off value of total score denoting oral squamous malignancy was then determined. Another 34 oral squamous malignancies that were misdiagnosed as non-malignant lesions on their pre-treatment biopsies were used as a validation cohort to test the clinical utility of this scoring system.

**Results:**

In the exploratory cohort, fifty-two (96%) of the 54 confirmed oral squamous malignancies had a total score of 9 and above. In the validation cohort, thirty-one (91%) of the 34 pre-treatment oral biopsy specimens also had a total score of 9 or above, supporting the feasibility of using this scoring system to predict immediate risk of oral squamous malignancy.

**Conclusions:**

Our four-biomarker “oral squamous malignancy scoring system” provides reliable prediction for immediate risk of oral squamous malignancy on pre-treatment oral biopsies.

**Supplementary Information:**

The online version contains supplementary material available at 10.1186/s12903-022-02188-0.

## Background

The mainstay treatment of oral cancer is surgery. Pathological confirmation remains to be the “gold standard” for diagnosis of oral squamous malignancy. However, according to the literature, a certain proportion of oral cancers was underdiagnosed on pre-treatment biopsies before curative surgery [1, 2], which could have resulted in suboptimal tumor resection. One important cause of equivocal diagnosis is due to inadequate sampling. The perplexing clinico-pathological features of oral verruco-papillary lesions (VPL) [3], such as oral verrucous hyperplasia (OVH) [4, 5], verrucous carcinoma (OVC) [6], and well-differentiated squamous cell carcinoma (OSCC), pose additional difficulties for the correct diagnosis of small oral biopsies [3, 7]. In addition, it is also not unexpected that routine pathological diagnosis based only on morphological features may have limitations in differentiating these challenging oral squamous lesions. Therefore, biomarkers indicating oral squamous malignancy will provide supplementary information to routine diagnostic pathology and facilitate the diagnosis of oral squamous malignancy on pre-treatment biopsies.

Abnormal differentiation, uncontrolled proliferation and tissue invasion/metastasis are the three most important characteristics denoting malignancy. Based on this idea, we hypothesized that, using representative immunohistochemical (IHC) markers to simultaneously evaluate these three important traits, we could confidently assess the biological similarity between an oral squamous lesion and a confirmed oral squamous malignancy, hence providing complimentary information facilitating the correct diagnosis of questionable biopsies. As a proof of idea, combining data from our previous study [8] and from literature research, we first selected IHC markers presumed to be representative for the three important biological traits of oral squamous malignancy. To accelerate translational application, we intentionally chose biomarkers that had been well explored in previous studies, with potential distinct expression patterns between non-malignant and malignant oral squamous lesions. Specifically, we chose cytokeratin 13 (CK13) and cytokeratin 17 (CK17) as markers for differentiation [9, 10], Ki-67 as a marker for proliferation [11, 12], and laminin-5 gamma 2 chain (Ln5γ2) as a marker representing tissue invasion/metastasis [13–16]. Although these biomarkers have been extensively studied, their translational utilities and/or limitations have not been clearly established.

Following marker selection, we explored the expression patterns of CK13, CK17, Ki-67 and Ln5γ2 on the surgical specimens in a discovery cohort of oral squamous malignancies. The distinct expression patterns of each biomarker were then categorized, scored, and combined to establish a scoring system. The scoring system was subsequently used to assess the immediate risk of malignancy for a given oral squamous lesion using another independent cohort of specimens. We validated that this new scoring system could be a useful tool to facilitate routine pathological workflow for the immediate diagnosis of oral malignancies in pre-treatment oral biopsies.

## Materials and methods

### Gene expression levels of KRT13, KRT17, Ki-67 and LAMC2 in non-tumor epithelia versus oral squamous cell carcinoma (OSCC)

The study protocol was reviewed and approved by the institutional review board (# B-ER-105–100). The gene expression levels of CK13, CK17, Ki-67 and Ln5γ2 (LACM2) between adjacent non-malignant oral epithelia and their corresponding OSCCs were compared using microarray data retrieved from our previous genome-wide association study using 40 paired OSCC samples [8], and data from The Cancer Genome Atlas (TCGA) database based on RNAseq technique. TCGA datasets were downloaded from Xena (https://xenabrowser.net/). TCGA-HNSC dataset contains 500 cases of head and neck squamous cell carcinoma (HNSC) tissues, with 44 of them having data from paired non-tumor epithelia. Among these 44 HNSC patients, only 19 of them were OSCC patients (TCGA-OSCC N/T pair cohort). All of the 40 OSCC patients in our array-based study [8] were betel quid users, while none of the 19 OSCC patients in the TCGA-OSCC N/T pair cohort was reported to use betel quid or areca nut.

### Pathological specimens

Oral verrucous carcinoma (OVC) is well recognized as a difficult diagnostic entity on pre-treatment biopsies [17]. In an attempt to address such difficulty, we first retrieved 33 formalin-fixed, paraffin-embedded (FFPE) archival OVC specimens to conduct this study. All of these 33 patients received curative surgery at the National Cheng Kung University Hospital during 2011–2019 and had pathologically confirmed OVC. Since synchronous/metachronous occurrence of SCC is a unique feature found in patients with OVC [17], we were able to obtain 21 OSCC specimens additionally from these patient. Thus, a total of 54 oral squamous malignancies (33 OVCs and 21 OSCCs) were used in the discovery cohort to explore the expression patterns of CK13, CK17, Ki-67 and Ln5γ2 in oral squamous malignancies. Seven papillary squamous cell carcinoma (PSCC) specimens were also included to demonstrate the expression patterns of these biomarkers in this unique OSCC variant.

To validate the clinical utility of the established scoring system as an adjuvant tool for assessing immediate risk of oral squamous malignancy, another independent cohort of oral biopsy specimens were used to evaluate the utility of this scoring system, including 11 benign oral biopsies containing non-malignant oral squamous epithelium (5 papilloma, 4 fibroma/fibroepithelial polyp, 1 Fordyce’s granule), 11 malignant biopsies (2 OVCs and 9 SCCs), and 34 non-malignant pre-treatment biopsies that were confirmed to be oral squamous malignancies (either OVC or OSCC) after surgical resection of the same lesions within 3 months following biopsy. Information regarding the clinical parameters, biopsy and surgical pathologies for the 34 patients were provided in Additional file 2: Table S1.

It was reported that some biomarkers, such as CK17 and Ln5γ2, could also be detected in non-malignant, regenerating epithelium such as wound healing [13, 18]. We further obtained specimens from diseases known to be associated with oral mucosal damage (1 ulcer, 4 lichen planus, 1 suprabasal bullous formation) to explore the limitation(s) for the usage of these biomarkers.

### Immunohistochemical (IHC) study

Briefly, sections of 4-µm thickness FFPE slides were first deparaffinized and rehydrated through graded alcohols. Heat-induced epitope retrieval (HIER) was performed by soaking slides in Target Retrieval Solution (DaKo, Glostrup, Denmark) (#S1699, PH 6.0 for CK17, Ki-67 and Ln5γ2; #S2367, PH 9.0 for CK13) using autoclave (120 ℃ for 20 min). After blocking endogenous peroxidase activity (Biocare's Peroxidazed 1, Biocare, CA, USA), the sections were then incubated with mouse anti-human CK13 (1 µg/ml, clone DE-K13, #sc-6258, Santa Cruz, Heidelberg, Germany), CK17 (2 µg/ml, clone E3, Abcam, MA, USA) or Ln5γ2 (2.5 µg/ml, clone D4B5, #MAB19652, EMD Millipore, CA, USA) at 4℃ overnight. Visualization was then performed with anti-mouse horseradish peroxidase (HRP)-conjugated polymer (Biocare), with 3,3’-Diaminobenzidine (Betazoid DAB) as chromogen (#BDB2004, Biocare). Mouse IgG1 (#X0931, DaKo) in a concentration similar to each specific antibody was used as an isotype control. Stained sections were then counterstained with hematoxylin and observed under light microscope. For Ki-67 staining (1 μg/ml, mouse monoclonal MIB-1, #M7460, DaKo), double staining of CK14 (1 μg/ml, rabbit polyclonal antibodies, ab15461, Abcam) was also performed to illustrate basal epithelial layer using MACH 2 Double Stain 2 kit (#MRCT525, Biocare). Color development of Ki-67 and CK14 was then performed using Betazoid DAB and Ferangi Blue (substrate of alkaline phosphatase, #FB813, Biocare) as chromogens, respectively. Stained sections were then counterstained with hematoxylin (CK13, CK17, Ln5γ2) or methyl green (Ki-67 and CK14, then observed under light microscope.

### Establishing a scoring system for assessing the immediate risk of oral squamous malignancy

**Fig. 1:**
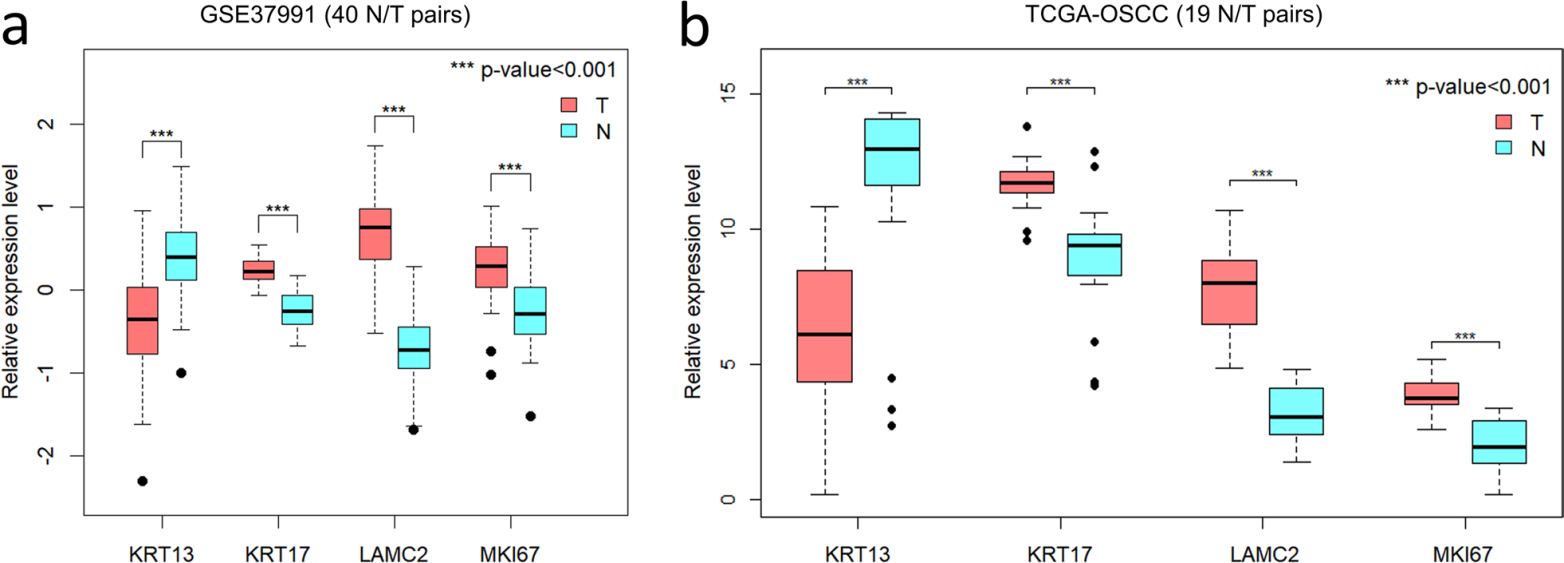
Gene expression levels of biomarkers in matched non-tumor epithelia and oral squamous cell carcinoma (OSCC). Compared to adjacent non-tumor epithelia (N), the corresponding tumor tissues (T) express significantly lower level of KRT13, while the expression of KRT17, LAMC2, and MKI67 are significantly upregulated in both (**a**) the betel-quid associated OSCC cohort (n = 40) in our study and in (**b**) The Cancer Genome Atlas (TCGA)-OSCC N/T pair cohort (n = 19)

Immunostained slides were evaluated under light microscopy. Each specific staining patterns of CK13, CK17, Ki-67 and Ln5γ2 was categorized based on their distinct expression patterns (Table 1, Fig. 1). A score was assigned to each specific staining pattern according to its relative importance in defining malignancy (detailed in the Discussion section). Briefly, the expression patterns of CK13, CK17, Ki-67 and Ln5γ2 in normal squamous epithelium were assigned to have the lowest score (score 0). Diffuse basal, with or without parabasal expression of Ki-67 was considered as a sign for uncontrolled proliferation and an important hallmark of malignancy (score 3). Cytoplasmic expression of Ln5γ2 in tumor cells located at the tumor-stromal interface was considered as a sign of tissue invasion [13] with a strong association of malignancy. Ln5γ2-positivity was thus designated to have the highest score (score 4) for potential oral malignancy. The total score for a given pathological specimen was then calculated by summing up the individual pattern-related score from CK13, CK17, Ki-67 and Ln5γ2, respectively. A cut-off total score was then determined and used to validate the usage of this scoring system to facilitate the assessment of immediate risk for oral malignancies in the validation cohort of oral biopsy specimens.

**Table 1:** Expression patterns of CK13, CK17, Ki-67 and Ln5γ2 in oral verrucous carcinoma (OVC) vs squamous cell carcinoma (OSCC)

Marker	Representative expression pattern		OVC (n = 33)		OSCC (n = 21)		*p value*	Pattern score
			n	(%)	n	(%)		
CK13	A	Strong expression from 1st parabasal (2nd basal) layer	0	(0)	0	(0)		0
	B	Decreased expression (other than pattern A or pattern C)	13	(39)	12	(57)	0.20	1
	C	Very weak of total loss of CK13 expression	20	(61)	9	(43)		2
CK17	A	Very weak or no expression of CK17	3	(9)	0	(0)		0
	B	Increased expression (other than pattern A or pattern C)	4	(12)	8	(38)	0.18^a^	1
	C	Strong expression at tumor-stromal interface or whole epithelial layers	26	(79)	13	(62)		2
Ki-67	A	1st parabasal layer ± scattered basal layer expression	0	(0)	0	(0)		0
	B	Predominant basal layer expression (peripheral expression)	11	(33)	15	(71)	0.06^b^	3
	C	Diffuse expression both in basal and parabasal layers	22	(67)	6	(29)		3
Ln5γ2	A	No expression (A1) or on intact basement membrane only (A2)	1	(3)	0	(0)		0
	B	Focal cytoplasmic staining cells at tumor-stroma interface (< 10%)	15	(45)	2	(10)		4
	C	Limited cytoplasmic staining cells at tumor-stroma interface (10–50%)	12	(36)	7	(33)		4
	D	Diffuse cytoplasmic staining cells at tumor-stroma interface (> 50%)	5	(15)	12	(57)	0.0012^c^	4

^a^CK17 Pattern C of OVC (79%, 26/33) versus CK17 Pattern C of OSCC (62%, 13/21)

^b^Ki-67 Pattern C of OVC (67%, 22/33) versus Ki-67 Pattern C of OSCC (29%, 6/21)

^c^Ln5γ2 Pattern D of OVC (15%, 5/33) versus Ln γ 5 Pattern D of OSCC (57%, 12/21)

### Statistical analysis

The gene expression data from our previous array-based study and the RNAseq data from TCGA were first log transformed during the process of normalization. T-tests were used to compare the differences for the gene expression levels of KRT13, KRT17, MKI67 and LAMC2 between OSCCs and their corresponding non-tumor epithelia. Chi-squared tests were used to compare the differences in the distributions of specific staining patterns from each IHC marker used in OVC and SCC. A probability level less than 0.05 (*p* < 0.05) denoted a statistical significance. Cohen’s kappa value was calculated for each biomarker to assess the inter-observer reliability on the 34 pre-treatment oral biopsy specimens from the validation cohort.

## Results

### Gene expression levels of KRT13, KRT17, Ki-67 and LAMC2 in OSCCs vs adjacent non-tumor epithelia

In South and South-East Asia, including Taiwan, betel quid/areca nut contributes to a large proportion of head and neck cancers, especially cancers arising from oral cavity [19]. Using data retrieved from our previous study [8], we demonstrated that the gene expression level of KRT13 was significantly down-regulated in OSCCs compared to their corresponding non-tumor epithelia, while the expression levels of KRT17, MKI67, and LAMC2 in OSCCs were significantly upregulated (Fig. 1a). To rule out the possibility that the observed biomarkers changes in our previous array-based study (n = 40, all of them were betel nut users) could be caused by betel quid usage or geographic/racial variation, we additionally performed analysis on the expression levels of these biomarkers using RNAseq data obtained from the TCGA-OSCC N/T pair cohort (n = 19). Similar changes in these biomarkers were also noted in the TCGA cohort (Fig. 1b), implying that these four genes may be robust biomarkers dysregulated in OSCC, regardless of betel quid usage or geographic/racial variation.

### Expression patterns of CK13, CK17, Ki-67 and Ln5γ2 proteins in non-malignant squamous epithelia, OVC, OSCC and PSCC

**Fig. 2:**
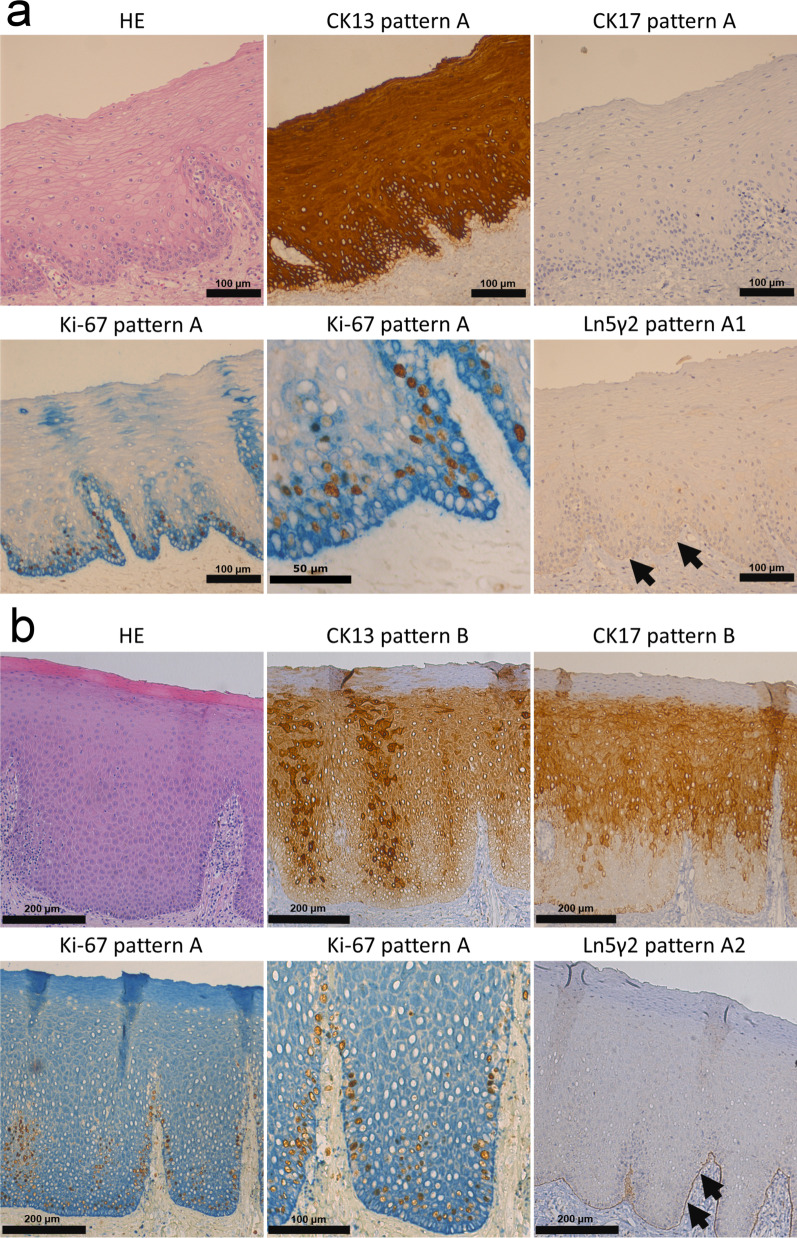
The expression patterns of biomarkers in non-tumor epithelia. **a** The histological picture of a nearly normal buccal mucosa (HE), showing a homogenous CK13 expression from the suprabasal layer (CK13 pattern A), absent CK17 expression (CK17 pattern B). The ki-67-labeled epithelial cells are confined to the suprabasal layers (Ki-67 pattern A). Ln5γ2 is barely detected at the junction between the epithelium and underlying connective tissue (Ln5γ2 pattern A1, arrow). **b** In an acanthotic epithelium (HE stain), decreased expression of CK13 is noted (CK13 pattern B), while expression of CK17 is increased (CK17 pattern B). The ki-67 positivity remains predominantly in the suprabasal layers (Ki-67 pattern A, magnified in the lower middle figure). A clear, uninterrupted staining of Ln5γ2 in the basement membrane is noted (Ln5γ2 pattern A2, arrows). HE, hematoxylin–eosin stain

Representative figures demonstrating each distinct IHC pattern of these four biomarkers are shown in Fig. 2. The distributions for each distinct IHC pattern from CK13, CK17, Ki-67 and Ln5γ2 in the 33 OVC and 21 OSCC specimens are shown in Table 1.

In non-dysplastic oral squamous epithelia (Fig. 2a), CK13 was intensely expressed from the 1st parabasal layer and above (CK13 pattern A), while CK17 was usually not expressed (CK17 pattern A). The proliferation marker Ki-67 was mainly detected in the 1st suprabasal layer (Ki-67 pattern A) with occasional basal layer expression. Ln5γ2 was barely detected in the basement membrane area (Ln5γ2 pattern A1).

In some non-tumor epithelia adjacent to oral malignancy (Fig. 2b), decreased CK13 expression was frequently noted (CK13 pattern B), with increased expression of CK17 (CK17 pattern B), as shown in a previous report [20]. The proliferation marker Ki-67 still located mainly at the 1st suprabasal layer, while Ln5γ2 was detected on the intact basement membrane (Ln5γ2 pattern A2).

**Fig. 3:**
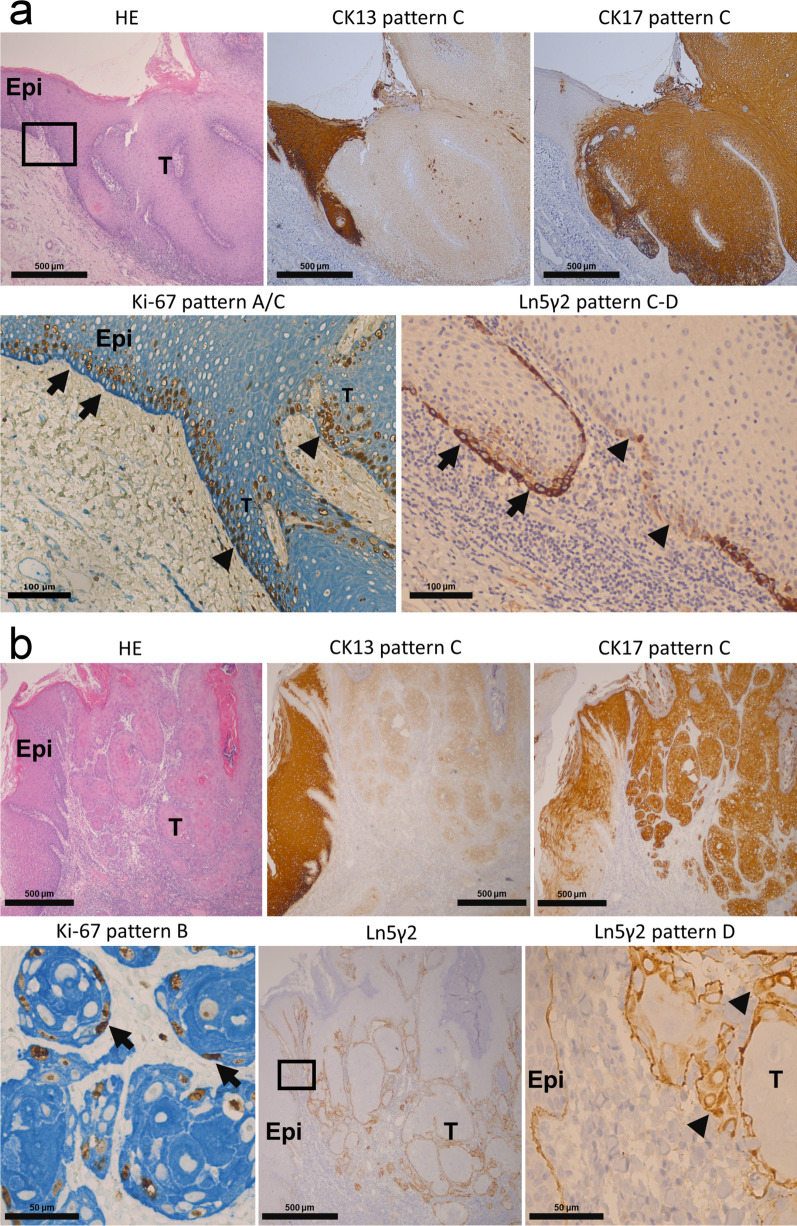
The expression patterns of biomarkers in oral verrucous carcinoma (OVC) and squamous cell carcinoma (OSCC). **a** A representative specimen of oral verrucous carcinoma (OVC) (HE). This tumor shows near-total loss of CK13 (CK13 pattern C), with intense CK17 in all tumor cells (CK17 pattern C). The expression pattern of Ki-67 in the adjacent non-tumor epithelium (Epi) is predominantly suprabasal (Ki-67 pattern A, arrows). In tumor tissues (T), the Ki-67-labeled cells are mainly detected in both basal and parabasal layers (Ki-67 pattern C, arrowheads). Cytoplasmic Ln5γ2 expression is expressed in tumor cells at the invasion front (Ln5γ2 pattern C, arrows), with an irregular, discontinuous basement membrane-like structure noted (arrowheads). **b** A representative specimen of oral squamous cell carcinoma (OSCC) (HE), with very weak expression of CK13 in tumor nests (T) (CK13 pattern C). Strong immune-reactivity of CK17 is also noted in the tumor nests (CK17 pattern C). Ki-67-labeled tumor cells are mainly detected in the basal layer (Ki-67 pattern B, arrows), with strong cytoplasmic staining of Ln5γ2 in almost all of the outer/peripheral tumor cells in the tumor nests (Ln5γ2 pattern D, arrowheads). HE, hematoxylin–eosin stain. Epi, non-tumor epithelia. T, tumor

In OVC (Fig. 3a), loss of CK13 was frequently observed (CK13 pattern C). In contrast, increased CK17 was noted in 91% (30/33) of OVCs, with 79% (26/33) of OVC specimens demonstrating intense CK17 staining in the tumor-stromal interface (CK17 pattern C). Ki-67 was predominantly located in the basal layer (Ki-67 pattern B), or diffusely expressed in both basal and parabasal layers (Ki-67 pattern C), with occasional extension into upper compartment above parabasal layers. Cytoplasmic Ln5γ2 expression was noted in tumor cells located at the tumor-stroma interface in 97% (32/33) of OVC specimens, with a discrete basement membrane-like structure. According to a previous study [13], the extent of Ln5γ2-positive cells at the tumor-stroma interface was further classified as no expression (0%, no Ln5γ2-positive cells noted), focal (< 10% Ln5γ2-positiev cells, Ln5γ2 pattern B), limited (10–50%, Ln5γ2 pattern C) or diffuse (> 50%, Ln5γ2 pattern D) expression in 3%, 45%, 36%, 15% of OVCs, respectively.

In OSCC (Fig. 3b), CK13 was significantly decreased or lost, as in OVC. Increased CK17 was noted in 100% (21/21) of OSCC specimens. Ki-67 was predominantly expressed in the basal cell layer on 71% (Ki-67 pattern B) of OSCC specimens, with 29% (6/21) of specimens showed diffuse staining in both basal and parabasal cell layers (29%, 6/21) (Ki-67 pattern C). Cytoplasmic Ln5γ2 expression in tumor cells was noted in all (100%, 21/21) of the OSCCs. The expression pattern of Ln5γ2 in OSCC was categorized as focal, limited or diffuse in 10%, 33%, 57% of OSCC specimens, respectively. The seven papillary SCC (PSCC) demonstrated similar expression patterns as OSCC (Additional file 1: Fig. S1).

Compared to most of the OSCC specimens that showed predominantly basal Ki-67 staining, OVC tended to have more diffuse Ki-67 expression in basal and parabasal layers (67% in OVC vs. 29% in OSCC, *p* = 0.06). In contrast, a significant higher proportion of OSCC specimens had diffuse expression pattern (pattern D) of Ln5γ2 when compared to OVC (57% in OSCC vs. 15% in OVC, *p* = 0.0012).

### Establishment of a scoring system for predicting oral squamous malignancies

According to the biological importance denoting malignancy, a pattern score was first assigned to each distinct IHC staining pattern of CK13, CK17, Ki-67 and Ln5γ2 (Table 1). For instance, invasion is considered as the most important biological consideration of malignancy. Thus, cytoplasmic expression of Ln5γ2 (Ln5γ2 pattern B, C, D) denoting invasiveness was assigned to have the highest pattern score (score 4), followed by markers representing uncontrolled proliferation (Ki-67 pattern B and pattern C, score 3), and markers representing impaired normal differentiation (CK13/CK17, pattern B and C, scored 1 and 2, respectively). A total score was then calculated by summing up the four individual pattern scores from CK13, CK17, Ki-67 and Ln5γ2. The distributions of total scores from oral squamous malignancies (33 OVC and 21 OSCC) and non-malignant oral squamous epithelia (n = 12) are shown in Table 2. Among the 54 oral malignancies, 96% (52/54) of them had a total score of 9 or above, indicating co-existence of all three malignant traits in these oral malignancies. Thus, we considered an oral squamous lesion with a total score of 9 and above as high risk for immediate oral malignancy that needs prompt intervention.

**Table 2:** Distributions of total score in various oral squamous lesions

Pathological specimens (n)			Total score (0–11) = pattern score (CK13 + CK17 + Ki-67 + Ln5γ2)											
			0	1	2	3	4	5	6	7	8	9	10	11
Cohort I	OVC	(33)							1		1	4	18	9
	OSCC	(21)										2	12	7
	PSCC	(7)										4	2	1
Cohort II	Papilloma	(5)					2		3					
	Fibroma/polyp	(4)	1	1					1	1				
	Fordyce’s disease	(1)	1											
	OVC	(2)											2	
	OSCC	(9)											5	4
	Oral biopsies	(34)		1				1			1	4	17	10

OVC, Oral verrucous carcinoma; OSCC, oral squamous cell carcinoma; PSCC, papillary squamous cell carcinoma

### Validating the scoring system as a complimentary tool for diagnosis of immediate risk of oral squamous malignancy

We subsequently used a cut-off total score of 9 as an indicator to predict immediate risk for oral squamous malignancy (Table 2). In the 11 benign oral lesions containing non-malignant oral squamous epithelium, all of them had a total score less than 9, while all of the 11 oral malignant biopsies had a total score of 9 and above. Compared to papillary SCC (Additional file 1: Fig. S1a), benign squamous papilloma consistently shows features favoring non-malignant squamous epithelia, such as intense CK13 expression, predominant 1st parabasal layer Ki-67 detection, and lack of cytoplasmic Ln5γ2-positivity (Additional file 1: Fig. S1b). Notably, in the 34 oral squamous malignancy that were misdiagnosed before surgical resection, thirty-one (91%, 31/34) of their corresponding pre-treatment biopsies had a total score of 9 or above, indicating immediate risk of oral squamous malignancy. To avoid subjectivity, the staining patterns of these four biomarkers from these 34 pre-treatment oral biopsies were also independently reviewed and scored by two observers (CLW and JRH, with clinical information blinded to CLW). The inter-observer reliability kappa values for CK13, CK17, Ki-67, and Ln5γ2 scoring were 0.83, 0.85, 0.87, and 0.85, respectively, indicating a strong level of agreement between observers. In addition, the two observers had 100% agreement on the benign vs malignant diagnosis in these 34 oral biopsy specimens.

It was reported that CK17 and Ln5γ2 might also be expressed during wound healing [13, 18]. To explore the potential limitations of our scoring system, six specimens from diseases known to be associated with oral mucosal damage (1 ulcer, 4 lichen planus, 1 suprabasal bullous formation) were used to decipher the expression patterns of these 4 biomarkers. Our results demonstrated that, in healing wound of oral mucosa (Additional file 1: Fig. S2a), CK 13 was evenly detected in whole layer at the advancing edge of the regenerating mucosa. Expression of CK17 was also increased, with basal CK17 expression noted in the adjacent, more organized epithelium. Ki-67 was irregularly detected at the advancing edge of the regenerating mucosa. Cytoplasmic staining of Ln5γ2 was noted in basal cells at the advancing edge, with a basement membrane-like structure continuous with the adjacent, more organized oral epithelium. In a representative specimen of lichen planus showing typical basal cell degeneration (Additional file 1: Fig. S2b), decreased expression of CK13 and increased expression of CK17 were noted. Ki-67 was mainly detected in the suprabasal layer above the degenerated basal layer. Ln5γ2 was similarly detected in the suprabasal layer, with an intact basement membrane below the degenerated basal layer. In the specimen with suprabasal bullous formation (Additional file 1: Fig. S2c), intense, suprabasal expression of CK13 was noted, as in normal mucosa. CK17 was expressed in the basal layer, similar to the regenerating mucosa. In contrast, compared to adjacent normal mucosa, Ki-67 was diffusely detected in the basal layer. Cytoplasmic Ln5γ2 was similarly detected in the suprabasal layer with an intact basement membrane. Taken together, our results demonstrated that some expression patterns of CK13, CK17, Ki-67 and Ln5γ2 mimicking oral squamous malignancy could also be detected on certain benign conditions associated with oral mucosal damage, indicating that these biomarkers should be used as a supplement, rather than a replacement, of routine pathological diagnosis.

## Discussion

The strength of our study lies in several aspects. First, based on a discovery cohort, we provided a comprehensive picture regarding the distinct staining patterns of biomarkers that represent the three most important biological characteristics (differentiation, proliferation and invasion/metastasis) for both non-malignant and malignant oral squamous lesions. With these unique patterns, we were able to reliably assess the biological similarity between a specific oral squamous lesion and confirmed oral squamous malignancies. The immediate risk for oral malignancy in a given biopsy specimen could thus be evaluated to facilitate clinical decision. Second, we used qualitative (pattern recognition), rather than quantitative approaches to score each distinct staining patterns of IHC markers. Therefore, our scoring system could be easily fitted into routine pathology workflow without the need of cumbersome marker quantitation or any expensive equipment. Third, since these biomarkers (CK13, CK17, Ki-67 and Ln5γ2) have been well explored in previous studies, the information obtained from previous studies provided useful background to consolidate translational usage.

For differentiation markers, intense, suprabasal expression of CK13 (designated as CK13 Pattern A in this study, score 0) was reported in normal, hyperplastic squamous epithelia [21] and oral squamous papilloma (OSP) [22]. In contrast, complete loss of CK13 (with or without focal activity in keratin pearls) (designated as CK13 Pattern C in our study, score 2) was noted in all [21], or almost all [9] of the OSCC specimens. Therefore, nonmalignant dysplastic squamous lesions [9, 21] with impaired squamous differentiation are more likely to have a CK13 expression pattern somewhere in between normal epithelia and OSCC (CK13 Pattern B in our study, score 1) (Fig. 2b). In contrast, oral malignant lesions, such as carcinoma in situ (CIS) or OVC, are more likely to have CK13 expression patterns similar to that of OSCC. Supporting this idea, loss of CK13 was reported in 93% of CIS [9] and 75% of OVC [22] in previous studies. We also demonstrated that complete CK13 loss was noted in 61% (20/33) of OVC and 43% (9/21) of OSCC (Table 1). For differentiation marker CK 17, same as in a previous study [20], we confirmed CK17 overexpression during oral carcinogenesis. Specifically, CK17 was not expressed in normal squamous epithelium (designated as CK17 Pattern A, score 0) (Fig. 2b), while increased CK17 expression was frequently detected in the adjacent non-cancer epithelia of OSCC (designated as CK17 Pattern B, score 1) [20]. Invasive nests in the stroma area of OSCC showed the highest intensity of CK17 staining (Fig. 3b) (CK17 Pattern C, score 2). We also noted intense CK 17 expression in more than 60% of OVC and OSCC specimens. A recent study also demonstrated that expression of CK17 could enhance proliferation and migration of OSCC cells by activating Akt/mTOR pathway [18], which provides a mechanistic explanation for CK17 upregulation in oral carcinogenesis.

Compared to abnormal differentiation, uncontrolled proliferation is another important hallmark of malignancy. Since the second basal cell layer (1st parabasal cell layer) is the proliferating center of normal oral mucosa, the proliferative marker Ki-67 was mainly detected at the 1^st^ parabasal layer in both normal and hyperplastic oral epithelia [11] (Ki-67 Pattern A in this study, score 0). During malignant progression from epithelial dysplasia to OSCC, Ki-67 was increasingly detected in the basal cell layer in almost 100% of CIS and OSCC [11]. Our study also showed that predominant basal Ki-67 expression (Ki-67 Pattern B), or diffuse basal and parabasal Ki-67 expression (Ki-67 Pattern C), was a consistent histological feature for both OVC and OSCC (score as 3).

Tissue invasion is the most convincing pathological feature denoting malignancy. However, inadequate sampling, or limited invasive feature of certain low-grade malignancies (such as OVC) may pose difficulty in pathological diagnosis on pre-treatment biopsies. Hence, surrogate maker(s) for tissue invasion will provide supplemental information to routine pathological examination based on hematoxylin and eosin (H&E) staining. Previous studies have demonstrated that cytoplasmic Ln5γ2 expression of in tumor cells located at the tumor-stroma interface is a consistent finding of OSCC [23, 24] and OVC [24]. We similarly confirmed that 100% of OSCC (21/21) and 97% (32/33) of OVC specimens did show cytoplasmic Ln5γ2 positivity in tumor cells (score 4 for Ln5γ2 pattern B, C and D) (Table 1). We also showed that OSCC was more likely to have a diffuse Ln5γ2 expression pattern (> 50%, pattern D) compared to OVC (57% in OSCC vs 15% in OVC, *p* = 0.0012), which is similar to a previous report demonstrating that 100% (15/15) of OSCC specimens had more than 5% Ln5γ2-positive cells (per 1000 counted cells), while all OVC specimens (15/15) had less than 5% of Ln5γ2-positive cells. Notably, in 20% (3/15) of the 15 OVC specimens, less than 1% of Ln5γ2-positive cells were detected [24].

There are various kinds of approaches in designing a scoring system. Our scoring system is based on biological considerations. We assumed invasion/metastasis as the most important biological trait associated with oral squamous malignancy, followed by uncontrolled proliferation and aberrant differentiation. Therefore, based on this idea, the differentiation score (CK13 and CK17) was assigned as 0 (normal, pattern A), 1 (pattern B), 2 (pattern C), while the proliferation score (Ki-67) was assigned as 0 (normal, pattern A), 3 (oncological proliferation pattern B, C, a score more than 2). The most important invasion/metastasis (Ln5γ2) score was assigned as 0 (no cytoplasmic expression, pattern A) and 4 (cytoplasmic expression, oncological pattern B, C, D, a score more than 3) (Table 1). Thus, a total score from these biomarkers would be able to estimate the biological similarity between an oral mucosa biopsy specimen and the estabolished oral squamous malignancies. Although a mathematical modeling was not used, it is evident that (Table 2) 96% (52/54) of OVC and OSCC (as well as all of the seven rare SCC variant, papillary SCCs) in the exploratory cohort had a total score equal or more than 9. A cut-off value of 9 was thus determined to denote immediate risk of oral malignancy. Validation of the reproducibility and pathobiology is the most important step to establish a clinical useful semi-quantitative scoring system [25]. The inter-rator reliability validated using an independent cohort of clinical samples supports clinical utility of this system.

The clinical usage of our scoring system deserves further attention. According to our scoring system, ninety-six percent (52/54) of the OVCs and OSCCs had a total score of 9 and above, implying the co-existence of all three malignant traits in these oral malignancies. Thus, a clinical suspicious biopsy with a total score of 9 and above should imply immediate risk of oral malignancy that needs prompt intervention. It is arguable that that Ln5γ2 may be used as a single biomarker to denote oral malignancy. However, since Ln5γ2 could also be expressed in certain non-malignant oral squamous diseases, supplemental markers for differentiation and proliferation (CK13, CK17 and Ki-67 in this study), as well as histological clues from routine hematoxylin and eosin (H&E) staining, are still mandatory to confidently imply malignancy. In addition, due to limited expression of Ln5γ2 in certain oral malignancy (such as OVC), a small biopsy specimen with inadequate representation of the entire lesion may fail to identify Ln5γ2-positivity. Thus, an oral biopsy specimen that is negative for Ln5γ2 expression but has typical patterns of abnormal differentiation and proliferation (hence with a total score of 5 and above) mimicking oral squamous malignancy, should still raise the alertness of potential malignancy. Re-sampling of the lesion or close follow-up is required.

Our study has several limitations. First, a plenty of premalignant squamous lesions were not included in this study. However, since the majority of these lesions were surgically removed, an outcome research to demonstrate clinical malignancy is not feasible. Therefore, we use only oral biopsies in which complete excision of the same lesion was performed within 3 months after biopsy to assess the immediate risk of oral malignancy. Thus, our scoring system could not be used to estimate the long-term risk of transformation in non-malignant oral squamous lesions. Second, we intentionally choose IHC markers that were reported to have well-explored expression patterns in previous studies. Therefore, novel markers unique for oral squamous malignancies could be overlooked. Third, although we did notice a few differential expression patterns (such as Ki-67 and Ln5γ2) between OVC and OSCC, the obtained information from our study was insufficient to differentiate OVC from OSCC. A plenty of biomarkers were reported to benefit the differential diagnosis of OVC from OSCC [7], however, the utility of these biomarkers remains to be validated. Fourth, we demonstrated that some non-malignant oral squamous lesions associated with mucosal damage could also have similar expression patterns of these four biomarkers seen in oral squamous malignancies, emphasizing the necessity of seeking additional biomarkers to differentiate these non-malignant oral mucosal lesions from oral squamous malignancies.

## Conclusions

In summary, by assessing the three important biological traits (differentiation, proliferation, and invasion/metastasis) denoting malignancy, we were able to establish a new scoring system to evaluate the biological similarity between an oral squamous lesion and oral squamous malignancies. Any suspicious oral squamous lesion with a total score of 9 and above should be highly considered as immediate risk for oral squamous malignancy. Our scoring system could be used as a supplement for routine pathological diagnosis that could benefit clinical decision-making.

## Supplementary Information

**Additional file 1**.** Figure S1 and S2**. Expression patterns of the four biomarkers in papillary SCC and various benign oral mucosal lesions.

**Additional file 2**.** Table S1**. The clinico-pathological parameters of the 34 patients with pre-treatment oral biopsy in the validation cohort (cohort II).

## Data Availability

All data generated or analyzed in this study is included in this published article and its Additional files 1 and 2.

## Abbreviations

OSCC: Oral squamous cell carcinoma
OVC: Oral verrucous carcinoma
HE: Hematoxylin and eosin (H&E)
IHC: Immunohistochemistry
