# Supplementary material for: A new scoring system facilitating diagnosis of oral squamous malignancy on biopsy specimens
Source: BMC Oral Health. 2022 May 6;22:165. doi: 10.1186/s12903-022-02188-0 (PMC9074340; doi:10.1186/s12903-022-02188-0)
Supplement: Supplementary file 1 — Additional file 1. Figure S1 and S2. Expression patterns of the four biomarkers in papillary SCC and various benign oral mucosal lesions. [file 12903_2022_2188_MOESM1_ESM.pdf]

A New Scoring System Facilitating Diagnosis of Oral Squamous Malignancy on Biopsy Specimens

Journal: BMC Oral Health

Cheng-Lin Wu[1,2], Cheng-Chih Huang[3], Shang-Yin Wu[2,4], Shih-Sheng Jiang[5], Fang-Yu Tsai[5], Jenn-Ren Hsiao[2,3]

1. Department of Pathology, National Cheng Kung University Hospital, College of Medicine, National Cheng Kung University, Tainan, Taiwan.
2. Institute of Clinical Medicine, College of Medicine, National Cheng Kung University, Tainan, Taiwan.
3. Department of Otolaryngology, National Cheng Kung University Hospital, College of Medicine, National Cheng Kung University, Tainan, Taiwan.
4. Department of Oncology, National Cheng Kung University Hospital, College of Medicine, National Cheng Kung University, Tainan, Taiwan.
5. National Institute of Cancer Research, National Health Research Institutes, Zhunan Town, Miaoli County, Taiwan

Correspondence to: Jenn-Ren Hsiao, MD, PhD
Department of Otolaryngology, National Cheng Kung University Hospital, College of Medicine, National Cheng Kung University
138 Sheng Li Road, Tainan 70456, Taiwan.
E-mail: hsiaojr@mail.ncku.edu.tw
Tel: 886-6-2353535 ext 5311; Fax: 886-6-2377404

Supplement Figure 1a

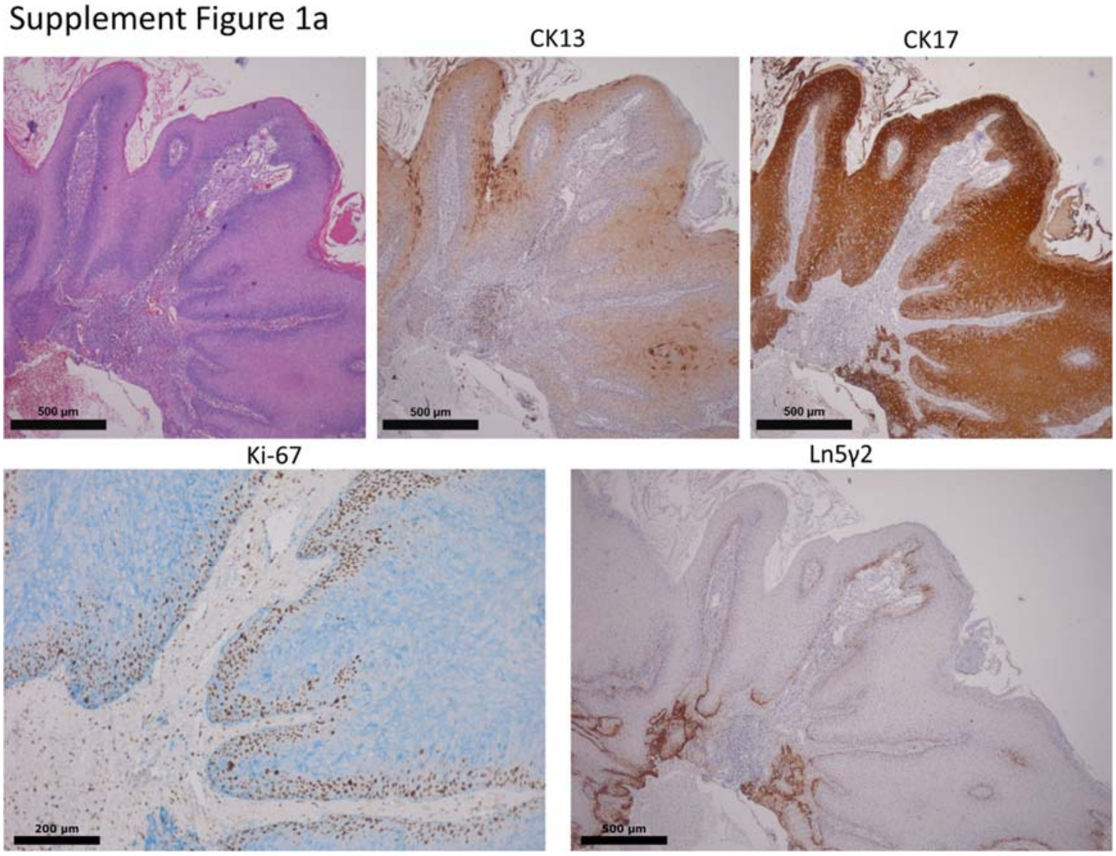

Supplement Figure 1b

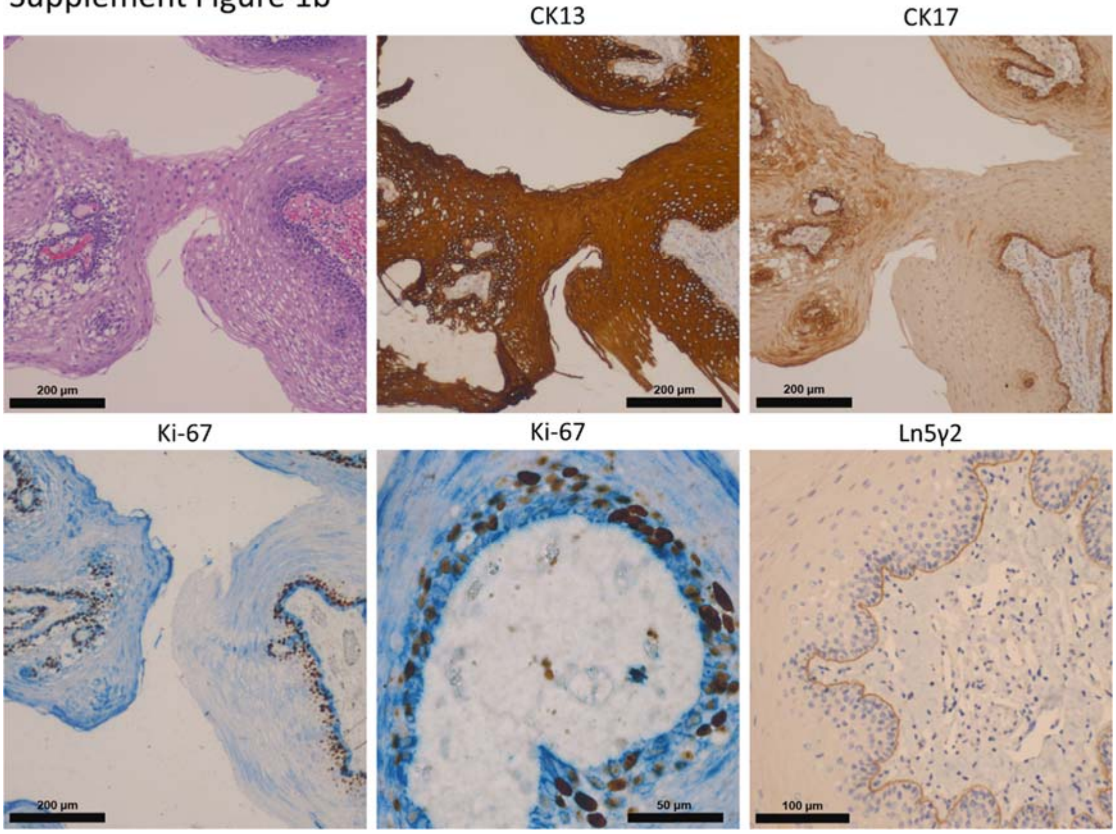

Supplement Fig.1

(a) A representative specimen of a papillary squamous cell carcinoma (HE). The lesion shows a papillary growth pattern with underlying invasion fronts. This tumor shows near-total loss of CK13 (pattern C), with strong immuno-reactivity of CK17 (pattern C). The Ki-67-labeled cells are diffusely distributed at both basal and suprabasal layers (pattern C). Ln5γ2 shows patchy staining pattern at the infiltrating borders (Ln5γ2 pattern B). Ln5γ2, laminin 5 gamma 2.

(b) In a typical squamous cell papilloma (HE), strong expression of CK13 is noted as in normal mucosa (pattern A), with weak CK17 expression in the suprabasal cells and moderate immuno-reactivity of CK17 in the basal layer (pattern B). The ki-67-positive keratinocytes are largely confined to the suprabasal layers (pattern A). Ln5γ2 staining reveals an intact and linear signal at the basement membrane area (pattern A)

Supplement Figure 2a

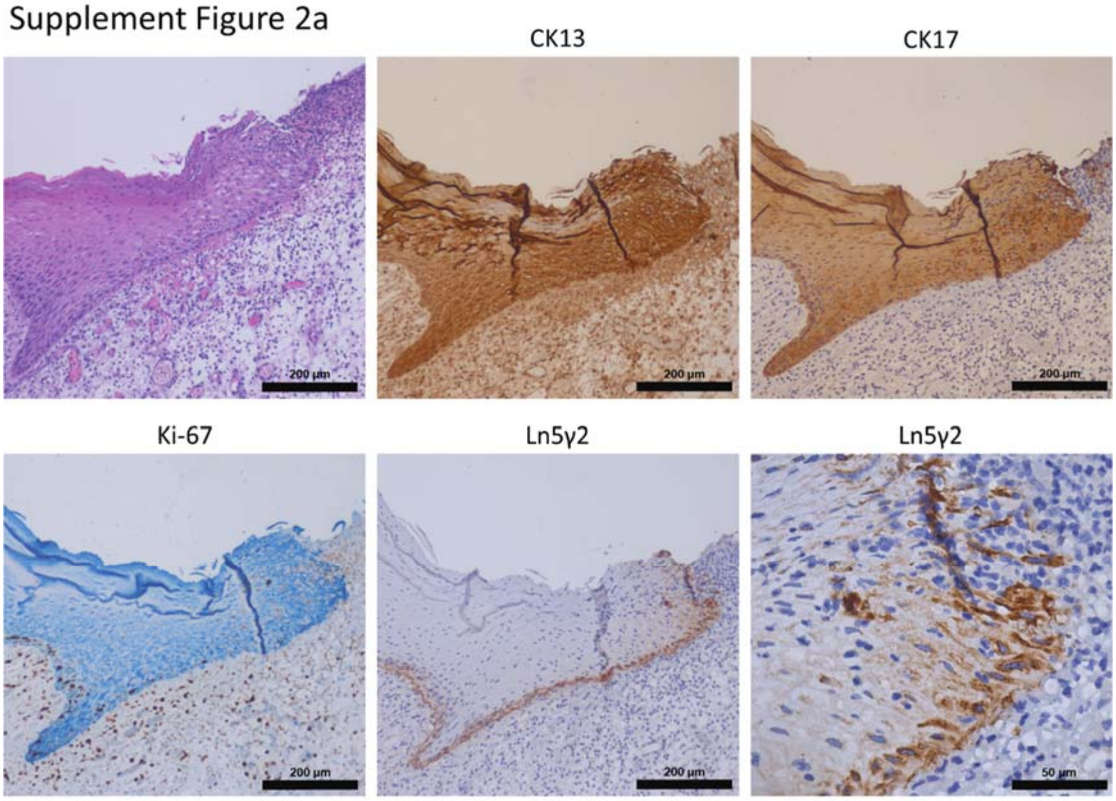

Supplement Figure 2b

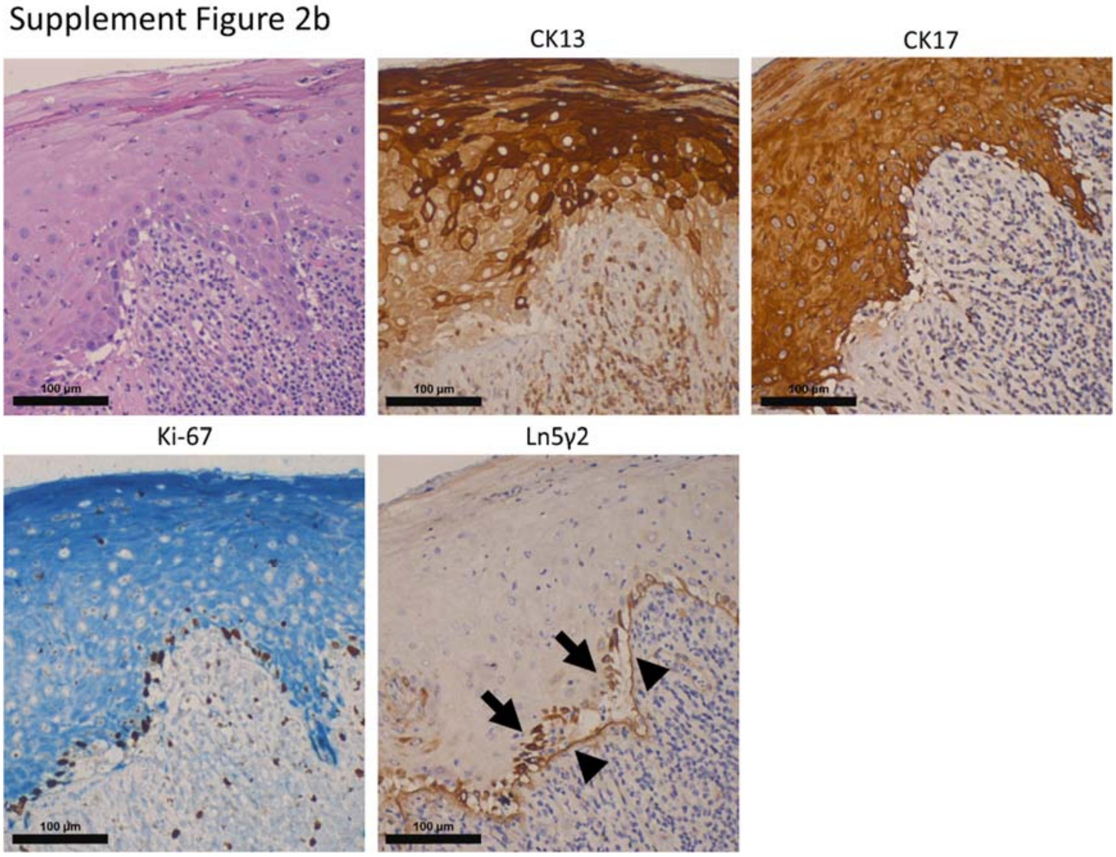

Supplement Figure 2c

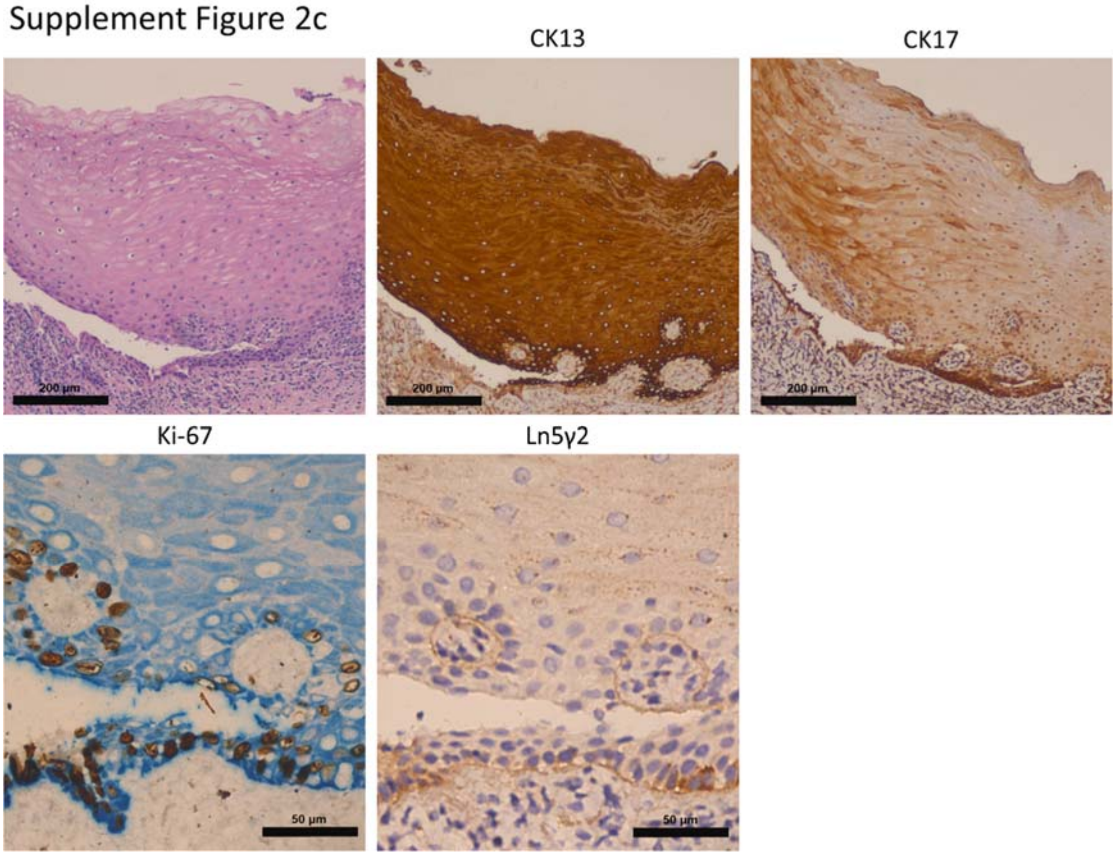

Supplement Fig. 2

(a) An oral ulcer specimen (HE). The regenerative epithelium at the periphery of an ulcer shows diffuse CK13 staining (pattern B) with increased CK17 immuno-reactivity (pattern B). Ki-67-positive basal keratinocytes are occasionally seen (pattern B). Increased Ln5γ2 expression is noted at the basement membrane area (pattern A2), with weak cytoplasmic staining of basal cells (Ln5γ2 pattern C). Ln5γ2, laminin 5 gamma 2

(b) A representative specimen of lichen planus, showing decreased CK13 expression (pattern B), with moderate immune-reactivity of CK17 (pattern B). Ki-67 staining cells was mainly detected at the suprabasal cells above the degenerated basal cell layer (pattern A). Diffuse cytoplasmic Ln5γ2 staining at both basal and parabasal cells are noted (arrows, pattern C), with a grossly intact basement membrane (arrow head, pattern A2)

(c) Representative pictures from a mucosal pemphigus lesion, which shows a well preserved CK13 expression as in normal mucosa (pattern A), with slightly increased CK17 expression (pattern B). Ki-67-positive cells are mainly seen in the basal layer (pattern B). Occasional cytoplasmic Ln5γ2 staining was also noted at basal cells (Ln5γ2 pattern B), with Ln5γ2 staining in the intact basement membrane (Ln5γ2, pattern A2)
