# Supplementary material for: A new scoring system facilitating diagnosis of oral squamous malignancy on biopsy specimens
Source: BMC Oral Health. 2022 May 6;22:165. doi: 10.1186/s12903-022-02188-0 (PMC9074340; doi:10.1186/s12903-022-02188-0)
Supplement: Supplementary file 2 — Additional file 2. Table S1. The clinico-pathological parameters of the 34 patients with pre-treatment oral biopsy in the validation cohort (cohort II). [file 12903_2022_2188_MOESM2_ESM.pdf]

Supplement Table 1. Clinical parameter, biopsy pathology and surgical pathology in the 34 patients with oral malignancy

Case No	Age	Sex	Subsite	Smoking	Drinking	Betel quid	Biopsy Pathology	Surgical Pathology
1	53	M	tongue	Y	Y	Y	moderate to severe dysplasia	SCC-MD
2	56	M	buccal	Y	Y	Y	moderate dysplasia	SCC
3	60	M	buccal	Y	Y	Y	papilloma	SCC
4	57	M	mouth floor	Y	Y	N	SH	SCC-MD
5	68	M	tongue	Y	Y	Y	papilloma	SCC-MD
6	63	M	buccal	Y	Y	Y	moderate dysplasia	SCC
7	51	M	palate	Y	Y	Y	hyperkeratosis	SCC-WD
8	41	M	buccal	Y	Y	Y	moderate dysplasia	SCC-MD
9	49	M	buccal	Y	Y	Y	SH	SCC-MD
10	49	M	buccal	Y	N	Y	SH with focal atypia	SCC-WD
11	64	M	gingiva	Y	Y	Y	SP	SCC-WD
12	48	M	buccal	Y	N	Y	VH	VC
13	43	M	buccal	Y	Y	Y	SH	SCC
14	45	M	buccal	N	Y	Y	SH	HC
15	50	M	buccal	Y	Y	Y	atypical epithelial hyperplasia	SCC-MD
16	51	M	mouth floor	Y	N	N	moderate to severe dysplasia	SCC-WD
17	56	M	buccal	Y	Y	Y	VH	VC
18	60	M	tongue	Y	Y	N	moderate dysplasia	SCC-WD
19	61	M	tongue	Y	Y	N	verrucous squamous proliferation	SCC-WD
20	79	M	buccal	N	N	N	VH	SCC-WD
21	61	M	palate	Y	Y	Y	SH	SCC-WD
22	50	M	buccal	Y	Y	Y	VH	VC
23	54	M	buccal	Y	Y	Y	VH	VC

24	79	M	buccal	Y	N	Y	SH	SCC-WD
25	67	M	tongue	Y	Y	N	SH	SCC-WD
26	71	M	mouth floor	Y	Y	Y	severe dysplasia	SCC-WD
27	62	M	gingiva	Y	N	Y	SP	SCC-WD
28	53	M	tongue	Y	Y	Y	atypical epithelial hyperplasia	SCC-WD
29	62	M	tongue	Y	Y	Y	SH	SCC-WD
30	72	M	gingiva	Y	Y	N	VH	SCC-WD
31	77	M	buccal	N	N	N	SH	SCC-WD
32	57	M	gingiva	Y	Y	Y	SP	SCC
33	52	M	tongue	Y	Y	Y	acanthosis	SCC-MD
34	40	M	lip	Y	Y	Y	atypical epithelial hyperplasia	SCC-WD

Abbreviations: SCC, squamous cell carcinoma; VC, verrucous carcinoma, HC, hybrid carcinoma; WD, well differentiated; MD, moderately differentiated; SP, squamous papilloma; SH, squamous hyperplasia; VH, verrucous hyperplasia.
